# Optimization of 3D ZnO brush-like nanorods for dye-sensitized solar cells†

**DOI:** 10.1039/c7ra13128c

**Published:** 2018-03-09

**Authors:** Simona Pace, Alessandro Resmini, Ilenia G. Tredici, Alessandro Soffientini, Xuan Li, Steve Dunn, Joe Briscoe, Umberto Anselmi-Tamburini

**Affiliations:** Department of Chemistry, University of Pavia Italy tau@unipv.it; School of Engineering and Materials Science and Materials Research Institute, Queen Mary University of London London UK j.briscoe@qmul.ac.ukc; Engineering and Technology, University of Hertfordshire College Lane Campus Hatfield AL10 9AB UK

## Abstract

In a dye-sensitized solar cell (DSSC) the amount of adsorbed dye on the photoanode surface is a key factor that must be maximized in order to obtain enhanced DSSC performance. In this study 3D ZnO nanostructures, named brush-like, are demonstrated as alternative photoanodes. In these structures, long ZnO nanorods are covered with a metal–organic precursor, known as a layered-hydroxide zinc salt (LHZS), which is subsequently converted to crystalline ZnO using two-step annealing. The LHZS is able to easily grow on any surface, such as the ZnO nanorod surface, without needing the assistance of a seed-layer. Brush-like structures synthesized using different citrate concentrations in the growth solutions and different annealing conditions are characterized and tested as DSSC photoanodes. The best-performing structure reported in this study was obtained using the highest citrate concentration (1.808 mM) and the lowest temperature annealing condition in an oxidative environment. Conversion efficiency as high as 1.95% was obtained when these brush-like structures were employed as DSSC photoanodes. These results are extremely promising for the implementation of these innovative structures in enhanced DSSCs, as well as in other applications that require the maximization of surface area exposed by ZnO or similar semiconductors, such as gas- or bio-sensing or photocatalysis.

## Abstract 2

Optimized 3D ZnO brush-like nanorods showing large surface area are presented as the photoanode in enhanced high-current-density DSSCs.

## Graphical abstract

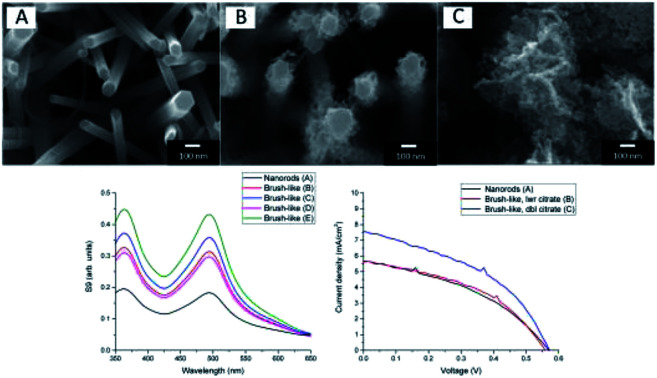

## 1 Introduction

In recent years, the development of new renewable sources of energy capable of replacing fossil fuels has been one of the most important problems in scientific and technological fields. Among all the possible types of renewable energies, solar energy appears very promising, mainly due to its accessible and nearly unlimited source of energy, the sun, and the absence of by-products.^1^ However, due to high costs of production of the commonly-used silicon-based solar cells, solar-cell technology is still not competitive enough to fully replace fossil fuel technology.^2^ In order to overcome these limitations, new types of solar cells have been proposed, and, among them, dye-sensitized solar cell (DSSC) technology is promising in terms of both low cost of production and high theoretical efficiency.^3^

The DSSC is a class of solar cell that is able to harvest solar energy by mimicking the photosynthesis process. In this solar cell, the photons are intercepted by a metal–organic dye, which is deposited on a wide bandgap semiconductor; then, if the incident photon has enough energy, a dye molecule is excited and a free electron is injected into the semiconductor and travels through it to the external circuit. The dye molecule is regenerated using a liquid electrolyte, usually I^−^/I_3_^−^; finally a platinum counter-electrode regenerates the electrolyte couple and closes the circuit.

In this cell, to obtain high performance, it is necessary that both the rate of the electron injection from the dye into the semiconductor and the rate of the electron transport from the semiconductor surface to the anode are higher than the rate of the recombination processes.^4,5^ To achieve this high performance and increase the percentage of injected electrons that reach the external circuit, each component of the DSSC needs to be optimized.

Among all the components, an optimized semiconductor material is of particular interest since both the amount of dye adsorbed and the probability that an excited electron reaches the external circuit depend on its structural and electronic properties.^6^ TiO_2_ is the material usually employed as photoanode in DSSCs^7^ and the highest efficiency reported so far is about 14%.^8^ In a DSSC, TiO_2_ is usually present as a nanoporous film whose large surface area enables a high concentration of adsorbed dye.^9–11^ However, when this nanostructure is employed, the electrons must pass across many grain boundaries; consequently, both the resistivity to their flow and the recombination probability increase.^12^ To improve DSSC efficiency, many different morphologies have been proposed in recent years, such as nanorods^13^ or nanowires.^14^ These 1D morphologies show high surface area on which the dye can adsorb, as well as a direct path for the electron flow from the semiconductor–dye interface to the external circuit. When these 1D morphologies are employed, the probability that the electrons reach the external circuit is increased,^15^ although the overall amount of exposed surface area is lowered. Despite the potential increase in DSSC efficiencies, obtaining these structures using TiO_2_ can be extremely difficult. Therefore, to overcome this issue, ZnO has been proposed as an alternative material in DSSCs.^3,16–19^ Compared to TiO_2_, ZnO presents a similar band gap, improved electronic properties, such as higher electron mobility, as well as the ability to grow in both 1D and 2D morphologies through simple techniques^20^ (*e.g.* hydrothermal growth).^21^

Although several studies have already described the properties of 1D and 2D ZnO,^22–25^ as well as their use as photoanodes in DSSCs, few studies have explored the use of more complex 3D ZnO structures in DSSCs. This lack of investigation might be due to the difficulty of nucleating a secondary ZnO structure on the surface of the nanorod. Generally, this secondary structure requires that the surface of the nanorod is covered with a new seed-layer, which is usually obtained by the immersion of the primary nanorod in a very dilute organic zinc salt solution and by the subsequent conversion of these seed-layers into ZnO nanoparticles by annealing.^26^ Despite the simplicity of this step, because of the low wettability of the surface of the primary nanorod to organic solvents, it is necessary to repeat this step several times in order to obtain a uniform and dense seed-layer on the surface of the primary nanorod. An alternative approach to fabricate 3D ZnO structures with enhanced exposed surface area employs a metal–organic precursor, named layered-hydroxide zinc salt (LHZS), that is able to easily nucleate on different types of surface,^27,28^ including the ZnO nanorod surface, without the assistance of a seed-layer. Using this method, the LHZS is homogenously deposited on the surface of the nanorods as a film of nanofoils. After the deposition, these nanofoils are annealed at low temperature so that the metal–organic phase is converted into ZnO nanofoils made of small homogeneous ZnO nanoparticles. This LHZS phase presents a layered brucite-like structure (Mg(OH)_2_) with the general formula M(OH)_*x*_^2−^(A^*m*−^)_*x*/*m*_·*n*H_2_O, in which M^2+^ is the metallic cation (in this case Zn^2+^) and A^*m*−^ is the organic counter-ion (*i.e.* nitrate, citrate or acetate). This structure consists of layers of Zn^2+^ cations, octahedrally coordinated by six hydroxyl anions, and interlayers of A^*m*−^ anions and water molecules, which are intercalated to balance the overall charge of the structure.^29^ Zhu *et al.*^28^ reported a similar strategy to increase the nanorod exposed surface area; however likely both the amount of nanofoils on the surface of the bare nanorods and the absence of subsequent annealing lead to DSSC overall efficiency equal to 0.67% when these 3D structures were employed. Therefore, a detailed study of the optimization of the preparation of this precursor, so that both the amount of exposed surface and the DSSC performance are maximized, is still required.

In this paper, the LHZS phase is employed as a ZnO precursor so that the surface of pre-existing ZnO nanorods can be easily covered with ZnO nanofoils, without the use of any intermediate seed-layer, to obtain a complex 3D structure, named brush-like. Additionally, the influence of the concentration of citrate in the growth solution, as well as temperature, time and atmosphere of the annealing on the morphology of the brush-like structure and the final DSSC performance are studied. As expected, when in the DSSCs bare ZnO nanorods are substituted with the brush-like structures, the conversion efficiency is improved and reaches 1.95% for the best sample tested.

## 2 Experimental section

### 2.1. Deposition of ZnO thin film

The ZnO seed-layer was prepared using a polymeric hydrogel precursor containing methanol, as solvent, Zn(NO_3_)_2_·6H_2_O, as cation source, 2,2-dimethoxy-2-phenylacetophenone, as photoinitiator, and poly(ethylene glycol)dimethacrylate (PEG-DMA, *M*_n_ 550), as oligomer. This organic precursor was prepared by mixing two intermediate solutions, A and B, so that the final concentrations of methanol, PEG-DMA and the photoinitiator were 43% wt, 43% wt and 8.4% wt respectively and Zn^2+^ was 0.3 M. Solution A was obtained by mixing 1 g of PEG-DMA, 0.5 g of methanol and 0.2 g of photoinitiator, whereas solution B contained 0.7 g of methanol and 1.5 g of zinc nitrate. The final solution was obtained by mixing 1 g of solution A with 0.35 ml of solution B. All the chemicals were purchased from Sigma-Aldrich and used as received. The substrates used in this work were 2 × 2 cm glass squares covered with FTO; before the growth these substrates were cleaned with acetone 99.8% and isopropanol, sonicating for 5 min and dried at room temperature.

Once the precursor solution was ready, 40 μl of this solution was dropped on the substrate and spin coated at 500 rpm for the first 10 seconds and then accelerated up to 1250 rpm for 35 seconds more. Then, the photopolymerization was activated under UV irradiation for 45 min using two UV lamps (*λ*_max_ 310 nm). Finally, the conversion to ZnO was obtained with thermal degradation in static air at 500 °C for 1 h.

### 2.2. Hydrothermal growth of ZnO nanorods

The nanorod growth solution was obtained by mixing Zn(NO_3_)_2_·6H_2_O (0.25 mM), ammonium hydroxide (650 mM), hexamethylenetetramine (HMT, 0.25 mM), polyethylenimine (PEI, 2.24 mM) and deionized (DI) water. All the chemicals were purchased from Sigma-Aldrich and used as received.

Once the solution was ready, the substrate loaded with the ZnO seed-layer described in Section 2.1 was immersed vertically against the wall of the bottle. Then this bottle was sealed and placed in an oven at 90 °C for 5 h. Finally, the sample was retrieved, rinsed with DI water and dried at room temperature.

### 2.3. Hydrothermal growth of ZnO brush-like structures

The LHZS growth solution was obtained by pouring under stirring Zn(NO_3_)_2_·6H_2_O (80 mM), HMT (80 mM), DI water and sodium citrate aqueous with final concentration of 0.904 mM (lower) or 1.808 mM (double). All the chemicals were purchased from Sigma-Aldrich and used as received. A ZnO nanorod sample was immersed in the solution and the hydrothermal treatment was carried out at 60 °C for 6 h. After the treatment, the sample was retrieved, rinsed with DI water and dried at room temperature. Finally, the conversion to ZnO nanoparticles was obtained by annealing for a first step at 350 °C for 1 h either in air or oxygen atmosphere. In addition to this, some samples underwent a second annealing step in air either at 400 °C for 10 min or at 500 °C for 30 min.

### 2.4. DSSC device fabrication

For the device fabrication, all the ZnO samples were immersed for 6 h in a 1 : 1 v/v acetonitrile : *tert*-butanol solution with N719 (0.3 mM) as dye-sensitizer and chenodeoxycholic acid (3 mM) as co-adsorbent. After removing and washing off the excess dye with ethanol, a 40 μm thick Sellotape film was used as a spacer to define the 5 × 5 mm active area. The cell was then closed with a platinum-based counter-electrode prepared using a platinum precursor (Platisol-T) on FTO. Finally, the liquid electrolyte Iodolyte HI-30 was added by capillarity force. All the chemicals were purchased from Solaronix and used as received.

### 2.5. Characterization

XRD measurements were performed using a Bruker D8 Advance diffractometer equipped with a Cu anticathode (*λ*_CuKα_ = 1.541838 Å) operating at 40 kV and 40 mA in theta–theta mode by using a step of 0.02 2*θ* degrees and a collection time of 30 s per step.

HR-SEM measurements were performed by using a TESCAN MIRA 3 XMU Microscope, operating at 30 kV on samples previously coated with a 5 nm Au layer (Cressington 208HR sputter). Cross-sections of the as-grown samples were prepared by cutting them in the bulk region using a diamond blade.

The dye loading on each sample was tested by recording the absorbance spectra of desorbed dye using a Perkin-Elmer Lambda 950 UV-visible spectroscope. Adsorbed dye was desorbed from the surface of the sample by soaking in 10 ml of a 0.1 M NaOH solution in 1 : 1 ethanol : DI water.

Finally, the photovoltaic performance of all DSSCs was measured using a solar simulator system (Newport 91160-100) with a SMU Keithley 2400 source meter to obtain the JV curve under 100 mW cm^−2^ (AM 1.5G) simulated sunlight.

## 3 Result and discussion

### 3.1. Structural characterization

First, ZnO nanorods were grown on an FTO substrate, which had been previously covered with a uniform film of ZnO seed-layer obtained using a hydrogel precursor. To grow these nanorods, the hydrothermal method was employed: the ZnO seed-layer was immersed in a growth solution in which ammonium hydroxide and PEI were added in addition to the common reactants HMT and zinc nitrate. When ammonium hydroxide is added to the growth solution, it has been shown to release NH_3,_ which then interacts with zinc ions and forms the complex [Zn(NH_3_)_*n*_]^2+^ (with *n* equals to 1, 2, 3 or 4). Because of the formation of this complex, ammonia acts as a Zn^2+^ ion buffer and maintains a constant zinc concentration in the solution, slightly higher than the limit of saturation for the heterogeneous precipitation and slightly lower than the limit of saturation for the homogenous precipitation. Therefore, the presence of ammonia suppresses the homogenous precipitation of ZnO and enhances the heterogeneous precipitation of ZnO nanorods along the [0001] direction and very long nanorods are obtained (details of this synthesis are reported elsewhere).^21^ In addition, the presence of PEI promotes the selective growth of thin ZnO nanorods along the polar direction [0001]. This is possible since the non-polar surfactant PEI tends to adsorb on the non-polar ZnO faces {0110}, {1100} and {1010} and to suppress the growth of the nanorods along the corresponding directions; consequently, the rate of the growth along the [0001] direction increases and the widening of the nanorod remains under control.

**Fig. 1:**
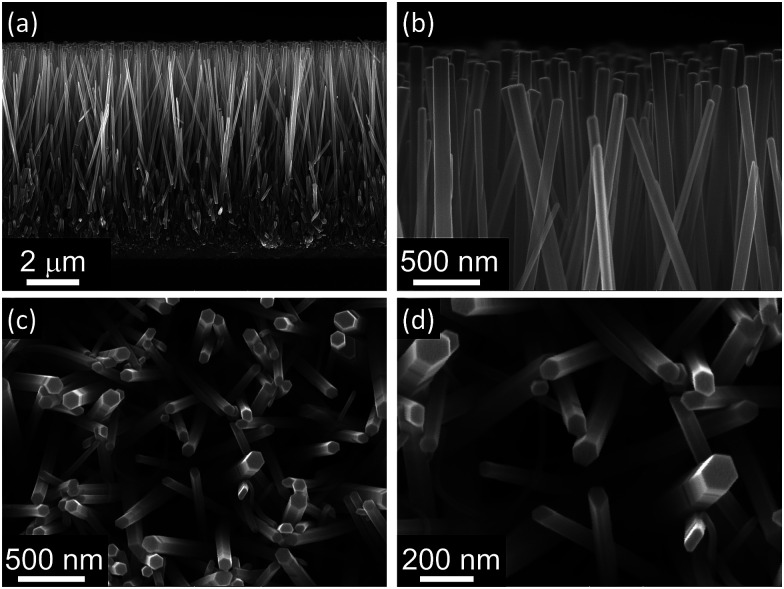
Cross-section (a and b) and top-view (c and d) SEM images of ZnO nanorods grown using the hydrothermal method. After heating at 90 °C for 5 h nanorods 8.6 μm long and 79 nm wide (aspect ratio 108) with a packing density of 29 nanorod per μm^2^ were obtained.

As a result of the addition of these two reactants to the growth solution, a dense film of ZnO nanorods 8.6 μm long and 79 nm wide (aspect ratio 108) with a packing density of 29 nanorod per μm^2^ was obtained after only 5 h of heating at 90 °C (Fig. 1). These nanorods also show high quality crystal structure with a preferential growth along the [0001] direction as shown in XRD analysis reported in a previous work.^30^

**Fig. 2:**
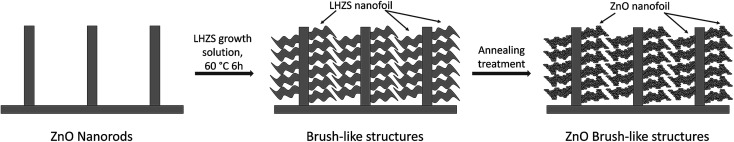
Schematic representation of the growth mechanism of brush-like structure. The ZnO nanorod are first covered with LHZS nanofoils; then the nanofoils are converted to ZnO nanofoils using annealing treatment.

3D brush-like ZnO structures were prepared by combining these nanorods with ZnO nanofoils obtained using a metal–organic precursor. Once the bare nanorods were prepared as described above, the secondary nanofoils were synthesized as described in the Methods. It was observed that during the treatment with the nanofoil growth solution an organometallic phase, known as layered-hydroxide zinc salt (LHZS), nucleates on the entire surface of the nanorods, while a ZnO phase homogenously precipitates. The organometallic phase grown on the surface of the nanorods must then be annealed to convert into ZnO nanofoils. In Fig. 2 a schematic representation of the growth of the brush-like structure is illustrated.

This annealing treatment must be performed at a temperature high enough to completely convert the metal–organic phase into crystalline ZnO but must not destroy the delicate nanofoil microstructure. Therefore, to find the best annealing conditions, preliminary work was carried out on powdered LHZS. It was clear from FTIR and XRD that the minimum temperature at which most of the organic phase was removed and the LHZS were fully converted in crystalline ZnO was between 350 °C and 400 °C (see ESI†). Thus, an intermediate annealing condition between those two temperatures (350 °C for 1 h plus 400 °C for 10 min) was chosen for this study in an attempt to retain the microstructure and therefore maximise the surface area.

**Fig. 3:**
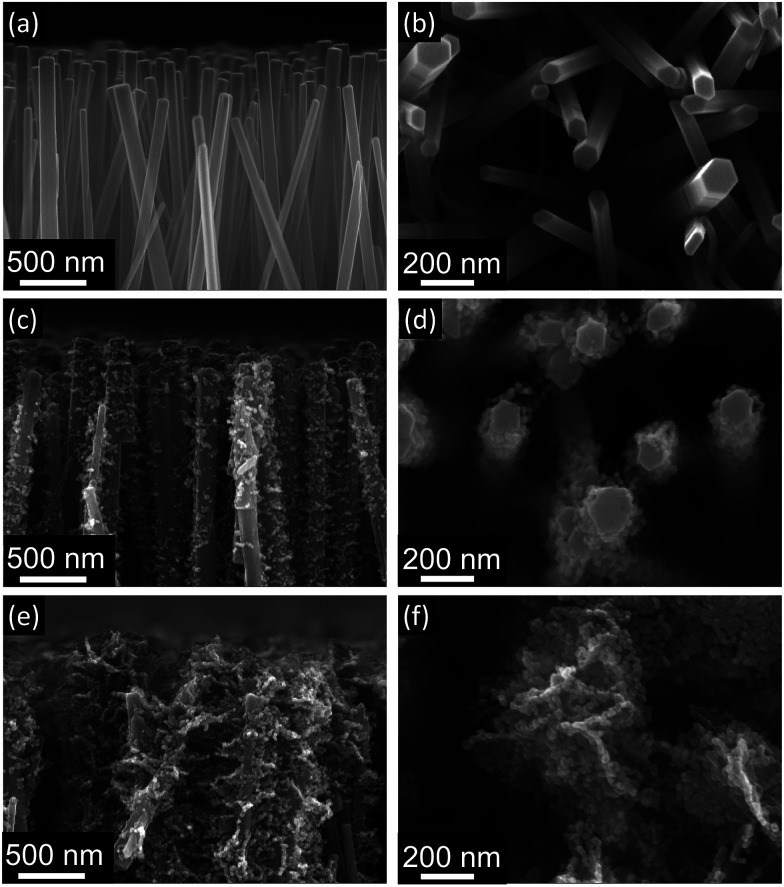
SEM images of bare nanorods (a and b) and brush-like structures grown using citrate concentration of 0.904 mM (c and d) and 1.808 mM (e and f), respectively. The increase of citrate concentration greatly increases the amount of nanofoils deposited on the surface of the nanorods.

Fig. 3 shows the morphology of nanorod and brush-like structures obtained using different citrate concentrations in the growth solution. It is evident that when a citrate concentration of 0.904 mM (Fig. 3c and d) was used, a small increase of exposed surface area was achieved compared to bare nanorods (Fig. 3a and b). Whereas, when the concentration of citrate ions in the growing solution was doubled from 0.904 mM (Fig. 3c and d) to 1.808 mM (Fig. 3e and f) and the same annealing parameters were used (350 °C for 1 h plus 400 °C for 10 min), the amount of ZnO nanofoils on the surface of the nanorods was greatly increased. In addition, it was noticed that when higher concentration of citrate was used, the turbidity of the growth solution decreased, suggesting that the homogeneous precipitation of ZnO in the solution decreases for higher concentration of citrate. This behaviour suggests that during the LHZS growth there is a competition between the homogeneous precipitation of ZnO and the heterogeneous precipitation of the LHZS phase. The images in Fig. 3 show that higher concentrations of citrate promote the deposition of LHZS phase. This result suggests that, during the growth, the citrate ions might act either as a catalyst for the heterogeneous deposition or as ligand of Zn^2+^ ions present in solution. The first effect would result in an increase in the LHZS deposition rate, while the second effect would control the concentration of free zinc ions in the solution, reducing the homogenous precipitation of ZnO. However, from preliminary results it was found that concentrations of citrate higher than 1.808 mM produces excess growth of the nanofoils on the ZnO nanorod surface, leading to fused structures that reduce, rather than increase the surface area.

### 3.2. DSSC performance

The brush-like structures discussed above were employed as photoanodes in DSSCs prepared using N719 as dye and iodine as liquid electrolyte. Their performance were compared with DSSCs employing bare nanorods as photoanodes. Table 1 summarizes the growth condition, the key DSSC performance and the maximum absorbance of desorbed dye from all the structures under study.

**Table 1:** Summary of grown parameters for each structure and related DSSC performance

Sample name	Structure	Citrate in growth solution (mM)	Annealing condition	Abs^a^	*J*_SC_ (mA cm^−2^)	*V*_oc_ (V)	FF	PCE (%)
A	Nanorod	—	—	0.195	5.0 ± 0.6	0.563 ± 0.006	0.41 ± 0.01	1.2 ± 0.1
B	Brush-like	0.904	350 °C_1 h + 400 °C_10 min	0.327	5.61 ± 0.02	0.547 ± 0.006	0.455 ± 0.002	1.39 ± 0.02
C	Brush-like	1.808	350 °C_1 h + 400 °C_10 min	0.372	7.49 ± 0.09	0.55 ± 0.02	0.452 ± 0.002	1.87 ± 0.06
D	Brush-like	1.808	350 °C_1 h + 500 °C_30 min	0.31	5.0 ± 0.2	0.52 ± 0.01	0.439 ± 0.009	1.13 ± 0.05
E	Brush-like	1.808	350 °C_1 h (oxygen)	0.448	7.97 ± 0.03	0.567 ± 0.006	0.433 ± 0.002	1.95 ± 0.03

a Absorbance value are obtained by taking the maximum of the UV-visible absorption spectrum of desorbed dye as shown in Fig. 5.

**Fig. 4:**
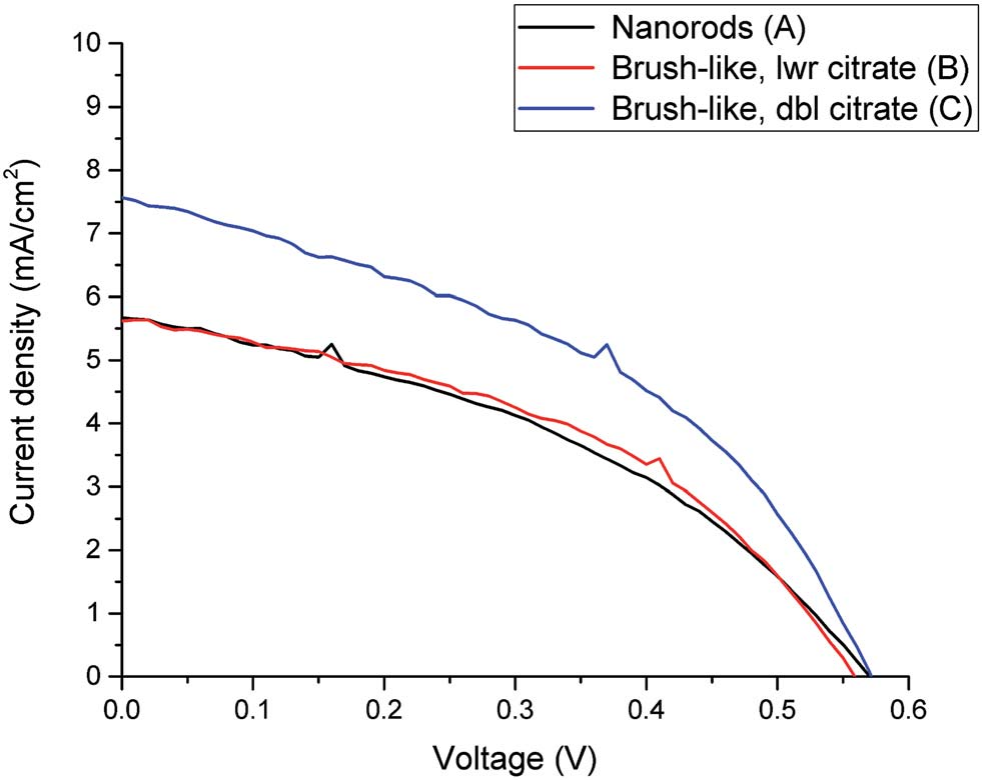
Current-density *vs.* voltage curves for DSSC made from nanorods (black line) and brush-like structures grown using lower (red line) and doubled (blue line) concentration of citrate.

Firstly, Fig. 4 compares the performance of DSSCs using photoanodes based on either nanorods or brush-like structures grown using different citrate concentrations but similar annealing conditions (samples A, B and C).

**Fig. 5:**
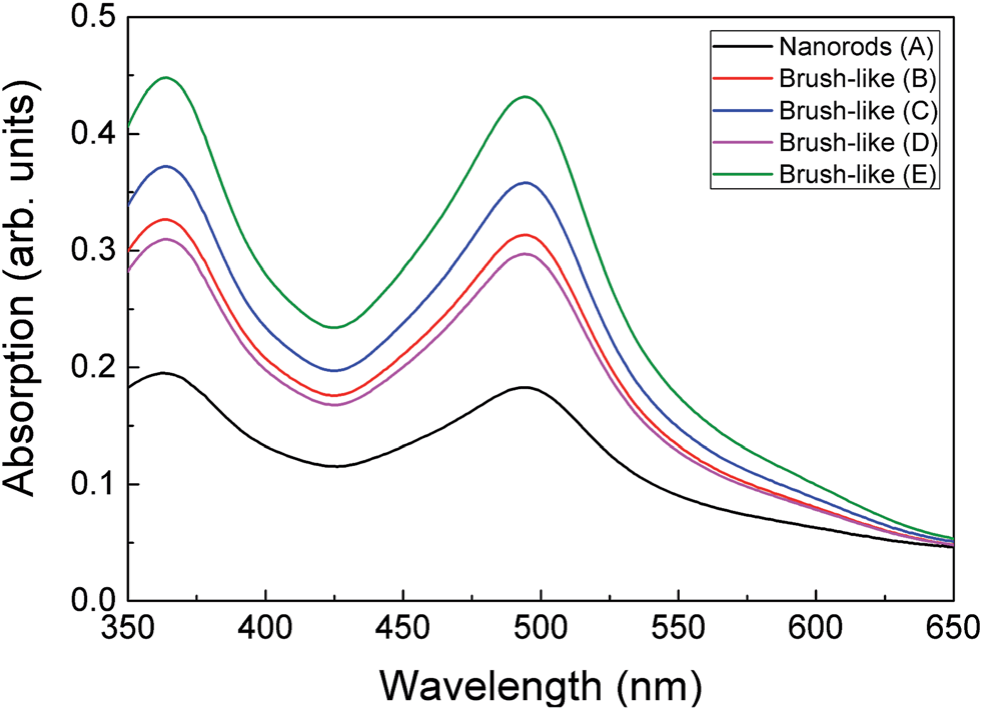
UV-visible absorption spectrum of dye desorbed from the surface of ZnO nanorods and brush-like structures, which were grown with different citrate concentrations and annealed in different condition. Sample preparation conditions are given in Table 1.

In Fig. 4 it is shown that if a small amount of nanofoils is grown on the surface of the nanorods, by using the lower citrate concentration (sample B), both the short circuit current density (*J*_SC_) and the power conversion efficiency (PCE) values only increase slightly compared to the bare nanorods (sample A). Thus, although when 1D nanorods are substituted with this 3D brush-like structures the amount of adsorbed dye on the surface increases (Fig. 5 and Table 1), the performance of the two DSSCs, and particularly *J*_SC_, does not change significantly. This implies that while the surface area for dye adsorption is increased by the addition of surface structures, other losses may be introduced which limit any enhancements in efficiency. This is discussed further below. However, when the citrate concentration is doubled to 1.808 mM (sample C) *J*_SC_ greatly increases from 5.0 ± 0.6 mA cm^−2^ for the bare nanorods to 7.49 ± 0.09 mA cm^−2^ for the brush-like structures obtained using doubled citrate concentration. When the citrate concentration is doubled, the amount of ZnO nanofoils on the surface of ZnO nanorods significantly increases, as shown in the SEM images (Fig. 3), thus the amount of dye adsorbed on this surface should increase consequently.

The relation between the increase of *J*_SC_ and amount of absorbed dye was further investigated using UV-visible analysis of dye desorbed from the surface of the structures (see Methods). Fig. 5 shows the optical absorption of the dye adsorbed onto ZnO nanorods and 3D brush-like structures, as well as further samples that are discussed below. As expected the absorption is found to be strictly dependent on the amount of surface area available on the different ZnO structures. In particular, the absorption of the brush-like structures is always significantly higher than the absorption of bare nanorods (sample A), regardless the specific preparation conditions used to obtain the 3D structures. In addition, when the brush-like structures are prepared using doubled citrate concentration (sample C) the absorbance increases strongly compared to the absorbance related to the structures prepared using lower citrate concentration and same annealing conditions (sample B). This increment in dye absorption and *J*_SC_ leads to a significant improvement of PCE, which raises from 1.2 ± 0.1% for nanorods to 1.87 ± 0.06% for brush-like structures grown using doubled citrate concentration (Table 1).

It is also notable that the fill factor (FF) for all brush-like structures (0.433–0.455) is in fact improved compared to the nanorod-based photoanode (0.41 ± 0.01), even though the structure of the photoanode changes from a monocrystalline one, such as ZnO nanorods, to a polycrystalline one, such as the ZnO brush-like structures. As discussed in the Introduction, DSSC efficiency depends not only on the amount of exposed surface area, but also on the path through which the electrons must travel to reach the external circuit. The nanofoils of these brush-like structures are made of ZnO nanoparticles; thus, when brush-like structures are used as photoanode, the electrons have to cross a great number of grain–grain boundaries to reach the external circuit. Despite this, the improvement in FF implies that recombination is lowered in the brush-like structure. This result suggests that the ZnO nanofoils produced from the LHZS structure may have improved properties for charge transfer from the dye, leading to reduced interfacial recombination that outweighs any increase in recombination at grain boundaries. Further detailed investigation of surface states and charge transfer kinetics at these different interfaces would provide further insight into this possibility, and would therefore be a potential avenue for future study.

To investigate whether reductions in the grain boundaries could improve the efficiency of the devices, photoanodes based on brush-like structures obtained using increased temperature and time in the second annealing step from 400 °C to 500 °C and from 10 min to 30 min respectively. This stronger annealing condition was chosen based on the preliminary FTIR results (see ESI†): when the annealing temperature reaches 500 °C, the organic phase is completely removed and the peaks related to the ZnO structure become more pronounced.

**Fig. 6:**
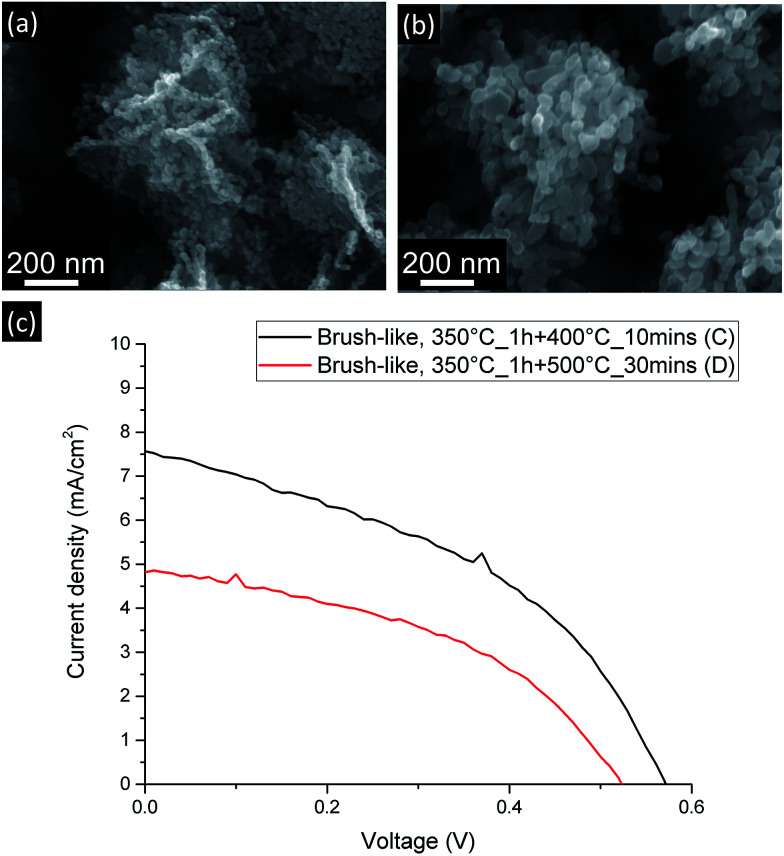
SEM (a and b) and current-density *vs.* voltage curves (c) for DSSC made from brush-like structures annealed using more moderate (a) and stronger (b) annealing conditions.

As shown in Fig. 6 (b), when the stronger annealing conditions are employed, the ZnO nanoparticles composing the nanofoils tend to sinter and grow and, consequently, both the number of grain–grain boundaries and the amount of exposed surface of these brush-like structures decrease.

As shown in Table 1 and Fig. 6 (c), *J*_SC_ greatly decreases from 7.49 ± 0.09 mA cm^−2^ down to 5.0 ± 0.2 mA cm^−2^ when the stronger annealing was used. The decrease is due to the decrease of dye absorption, which can be observed in the UV-visible absorption results (Fig. 5). Of particular interest is that when the brush-like structure underwent annealing at higher temperature and time (sample D), the absorption is even lower than that obtained using brush-like structure grown in lower concentration of citrate (sample B). This confirms that annealing at high temperatures for long times results in a degradation of the nanofoil microstructure producing only a small increase in the amount of surface area compared to bare nanorods. As a result, the PCE of sample D is much lower (1.13 ± 0.05%) than the one obtained using softer annealing (sample C, 1.87 ± 0.06%).

It is, in fact, interesting to note that even if the number of grain–grain boundaries is much lower in sample D because of the stronger annealing conditions employed, there is no improvement in the FF compared to the values of sample C (0.452 ± 0.002). This lack of increase means that even if the nature of the electron path is an important factor in DSSC optimization, in this case the amount of exposed surface area appears to be much more influential, thus any benefit gained by reducing the resistivity of the electron path is eclipsed by the decrease of ZnO exposed surface area.

**Fig. 7:**
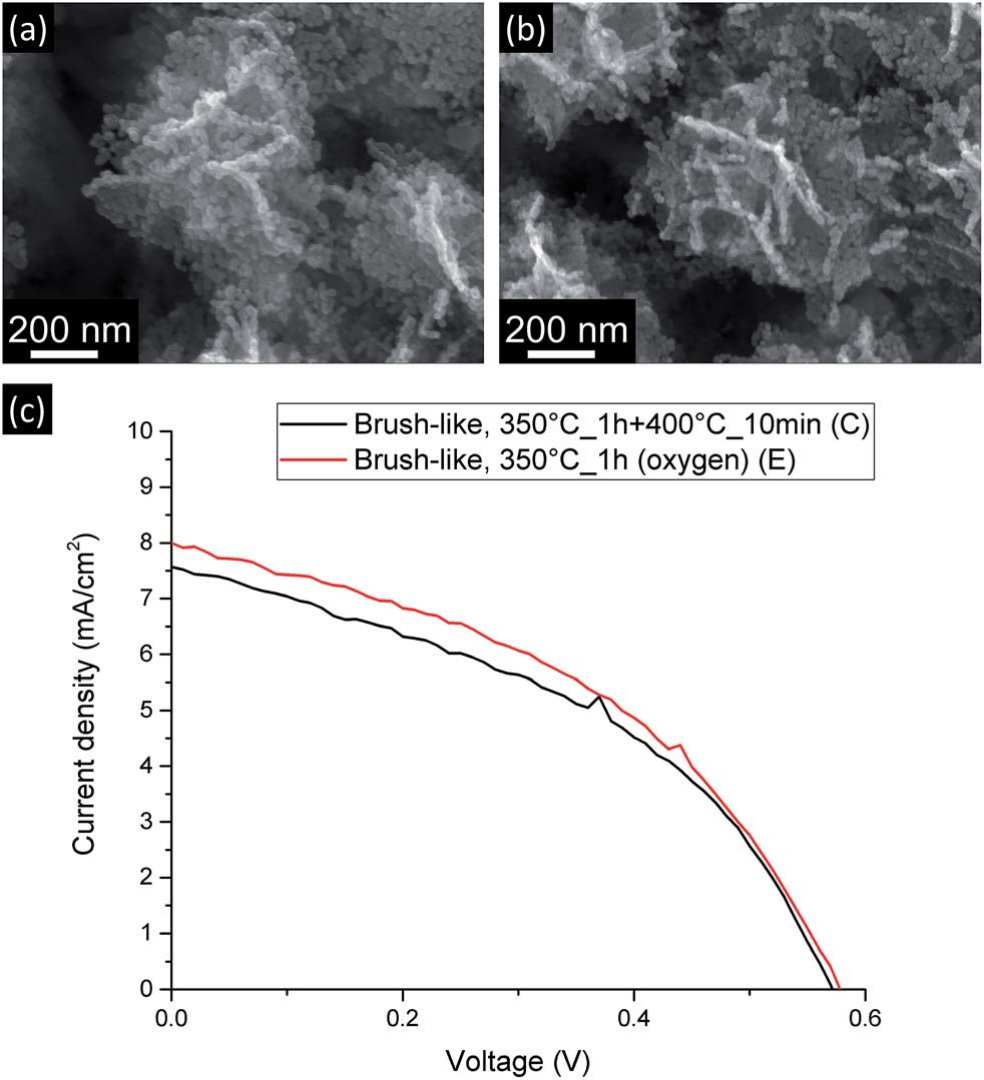
SEM images (a and b) and current-density *vs.* voltage curves (c) for DSSC made from brush-like structures annealed using 2-step annealing (a) and 1-step (oxygen atmosphere) (b).

Since the amount of exposed surface area has such a strong influence on the final DSSC performance, attempts were made to further decrease the annealing temperature at which the brush-like structures were converted. In this case only the first annealing step was used (350 °C for 1 h). However, as shown in the FTIR results (see ESI†), after this annealing step most of the organic phase was still present, thus, in order to overcome this limitation and to facilitate the oxidation of this phase, an oxidative environment was used during this annealing (pure O_2_). This was referred to as sample E. Fig. 7 shows the JV curves (c) and the SEM images (a and b) of these brush-like structures (sample E) and the structures obtained annealing in air using the second step at 400 °C for 10 min (sample C).

The brush-like structures annealed in oxygen (sample E) showed a small *J*_SC_ increase from 7.49 ± 0.09 mA cm^−2^ to 7.97 ± 0.03 mA cm^−2^, despite the large improvement of exposed surface area and dye absorption (Fig. 5). These results might imply that, despite the increased surface area obtained by reducing the annealing temperature (as shown by absorption spectra: Fig. 5), this was not translated in an equivalent improvement of *J*_SC_ in the DSSC device. It is likely that this discrepancy is due to an increase of recombination probability of electrons from the surface of the structure to the external circuit. These recombination events might be caused either by a great increase of the number of grain boundaries or by the presence of oxygen-related defects introduced by the annealing atmosphere. Again, detailed investigation of such processes would be an interesting area for future study.

Therefore, when a lower annealing temperature is employed, in combination with the use of oxygen atmosphere, the DSSC performance improves slightly, leading to an overall PCE equal to 1.95 ± 0.03%, but the improvement is lower than expected based on the increased dye loading (Fig. 5).

**Fig. 8:**
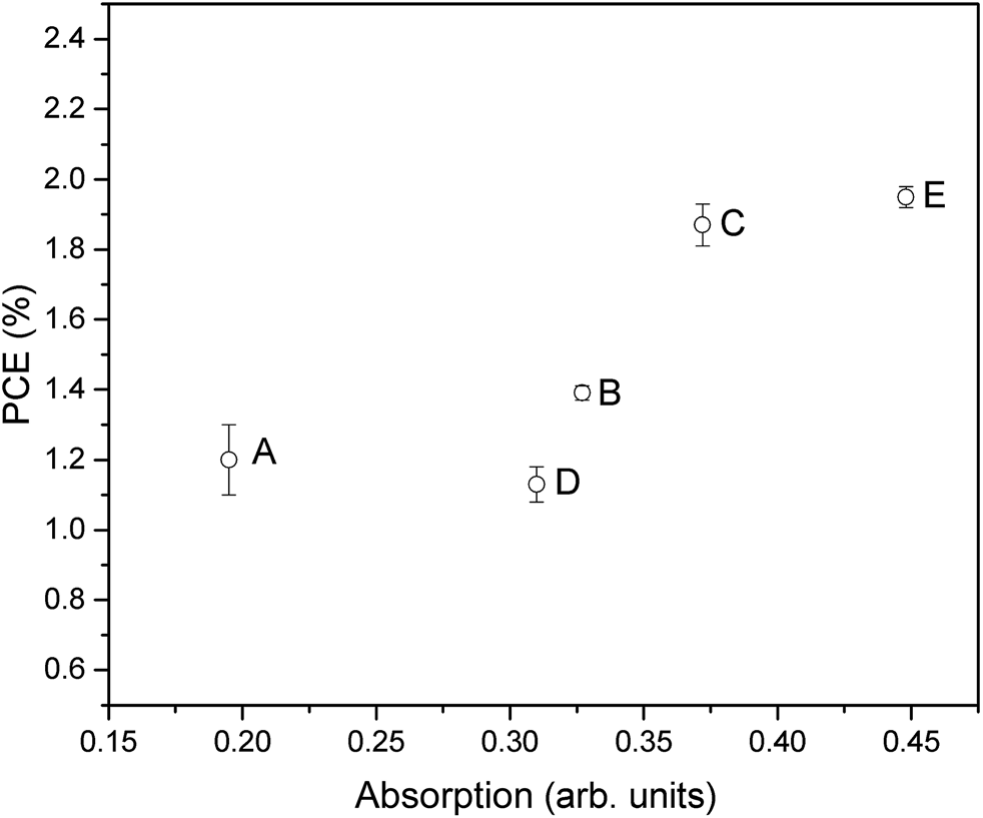
Optical absorbance *vs.* PCE plot showing the relationship between the amount of exposed surface area and the final DSSC performance.

By comparing the PCE of the DSSCs with the optical absorption of the desorbed dye (Fig. 8), it can be seen that in general these two parameters correlate well. This is to be expected since, as stated, the amount of surface area available and therefore amount of dye adsorbed onto the surface is an important factor in determining the PCE by influencing the light absorption and therefore *J*_SC_. However, it is also interesting to note that some samples show slightly lower PCE than expected from the UV-visible absorption, such as when higher temperature and time annealing was used (sample D), or when an oxygen annealing atmosphere was used (sample E). This highlights that further factors beyond surface area and dye adsorption can be influenced by the annealing such as surface states and therefore charge transfer and recombination processes as already discussed. However, in general this does highlight that given the optimum annealing conditions, the efforts discussed here to maximize available surface area through careful deposition and treatment of surface structures can be very beneficial for increasing the available surface area for dye adsorption and therefore PCE.

## 4 Conclusion

In this study 3D ZnO structures, named brush-like, have been proposed as an alternative DSSC photoanode material. These innovative structures were prepared by covering the surface of ZnO nanorods with nanofoils deposited using layered-hydroxide zinc salt (LHZS) as a metal–organic precursor. This metal–organic precursor was then converted in ZnO nanofoils using two-step annealing. When bare ZnO nanorods were substituted with these 3D structures as a DSSC photoanode, a consistent increase of both *J*_SC_ and PCE have been reported, up to maximum values of 7.97 ± 0.03 mA cm^−2^ and 1.95 ± 0.03% respectively. This enhanced performance is the result of the systematic optimization of the brush-like structure preparation process. First it has been found that higher concentrations of citrate in the growth solution promote the deposition of the LHZS phase at the expense of the homogenous precipitation. Therefore, as shown in SEM images, when higher concentrations of citrate were employed, larger amount of exposed surface area was obtained. This result agrees with both UV-visible analysis and DSSC test results, which show higher optical absorption (0.37) and higher PCE (1.87%) when higher citrate concentrations were used in the growth solution. In addition, the annealing parameters were optimized. This annealing step is necessary to convert the LHZS phase in fully-crystalline ZnO nanoparticles without destroying the nanofoil arrangement. It has been found that performing this annealing step in pure oxygen at 350 °C for only 1 h was sufficient to fully convert the LHZS phase in ZnO and obtain the highest DSSC performance reported in this study (absorption 0.45; PCE 1.95%).

These results suggest that these innovative 3D ZnO structures are a promising substitute for common 1D ZnO structures, such as nanorods, as a photoanode. When these brush-like structures are employed as DSSC photoanodes the *J*_SC_ dramatically increases due to the large amount of surface area exposed by these structures. In addition, it has been observed that even if the electron path from the adsorbed dye to the external circuit presents a large number of grain–grain boundaries, the FF values of these 3D structures are comparable to those of the bare nanorods. Therefore, when these structures are employed the probability of recombination processes does not appear to increase. However, despite these improvements the maximum efficiency remains below 2%, where efficiencies of up to 10% are achievable using similar dye-electrolyte systems in combination with mesoporous TiO_2_ photoanodes.^7^ Considering that maximum efficiencies similar to the ones achieved in this work are reported in related literature on ZnO nanostructures in DSSCs,^3,16–19,22–25^ this supports the conclusion that there are fundamental limitations to the use of ZnO nanostructures in DSSCs. Despite this, the overall findings reported herein – that through careful control of processing conditions an optimum hierarchical nanostructure can be produced with a careful balance between optimised surface area and conductive pathways – are highly relevant beyond this single application. The ability to directly observe the impact of these conditions on both dye adsorption and device performance parameters make the study of DSSCs extremely useful tool to understand this balance. The optimised structures identified through this development may, however, offer the greatest advantages when applied to wider fields where high surface area and good charge transport are also required, such as photoelectrocatalysis, and gas- or bio-sensing.

## Conflicts of interest

There are no conflicts to declare.

## Supplementary Material

RA-008-C7RA13128C-s001
