# Supplementary material for: Optimization of 3D ZnO brush-like nanorods for dye-sensitized solar cells
Source: RSC Adv. 2018 Mar 9;8(18):9775–82. doi: 10.1039/c7ra13128c (PMC9078819; doi:10.1039/c7ra13128c)
Supplement: RA-008-C7RA13128C-s001 [file RA-008-C7RA13128C-s001.pdf]

Electronic Supplementary Material (ESI) for RSC Advances.
This journal is © The Royal Society of Chemistry 2018

Optimization of 3D ZnO Brush-like nanorods for dye-sensitized solar cells

Simona Pace,[1] Alessandro Resmini,[1] Ilenia G. Tredici,[1] Alessandro Soffientini,[1] Xuan Li,[2] Steve Dunn,[2] Joe Briscoe[2] and Umberto Anselmi-Tamburini[1]

[1] Department of Chemistry, University of Pavia, Italy
tau@unipv.it

[2] School of Engineering and Materials Science and Materials Research Institute, Queen Mary University of London, London, UK. j.briscoe@qmul.ac.uk

Supplementary data

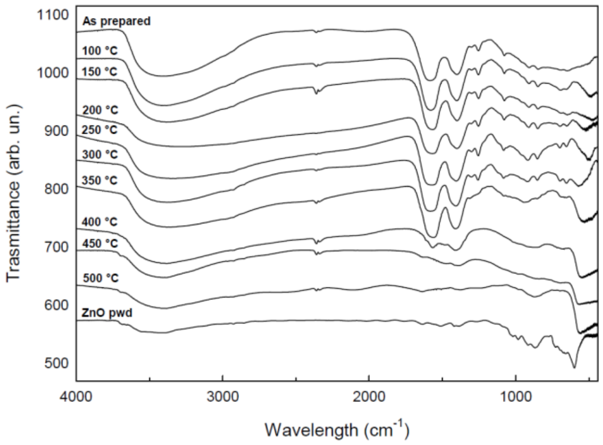

Figure S1. With the aim to determinate the lowest temperature necessary to remove most of the organic phase during the LHZS conversion in crystalline ZnO preliminary analysis on LHZS powder were carried out. Figure S1 shows the FTIR results for LHZS powder as prepared and annealed at different temperatures, ranging from 100 °C to 500 °C; in addition, the FTIR spectra of commercial ZnO powder is shown for reference. It is evident from this comparison how most of the organic phase, whose peaks lie at 1400 and 1570 cm^{-1}, is removed when the LHZS are annealed between 350 and 400 °C.

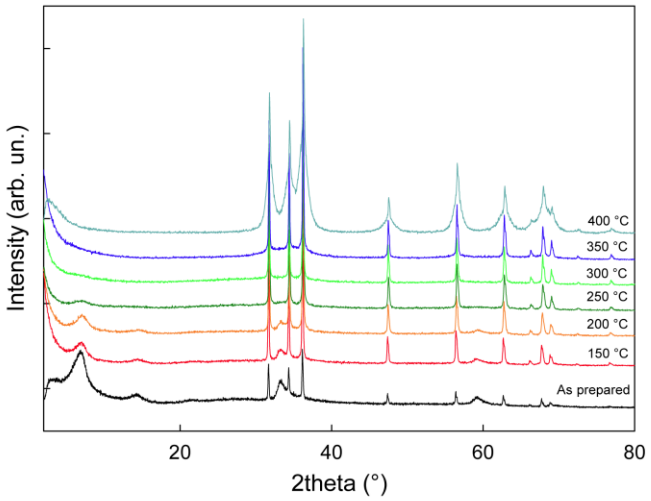

Figure S2 The XRD of powder LHZS as prepared and annealed at temperatures ranging from 150 °C to 400 °C. This structural analysis was employed in order to find the best annealing conditions to convert the metalorganic LHZS phase is crystalline ZnO without destroy the nanofoils structure via solid diffusion. As shown in Figure S2, the peaks related to the LHZS phase at 6.47, 14.1 and 33.29 ° 2θ disappear when the annealing temperature is equal to both 350 °C and 400 °C. Thus, this range of temperature must be reached to obtain crystalline ZnO nanoparticles from the metalorganic LHZS precursor.
